# Transcutaneous electrical acupoint stimulation for pregnancy outcomes in women undergoing *in vitro* fertilization-embryo transfer: A systematic review and meta-analysis

**DOI:** 10.3389/fpubh.2022.892973

**Published:** 2022-08-11

**Authors:** Han Yang, Wen-hui Hu, Gui-xing Xu, Zi-han Yin, Si-yi Yu, Jia-jia Liu, Zhi-yong Xiao, Xiao-yan Zheng, Jie Yang, Fan-rong Liang

**Affiliations:** ^1^Acupuncture and Tuina School, Chengdu University of Traditional Chinese Medicine, Chengdu, China; ^2^Chengdu Xinan Gynecological Hospital, Chengdu, China

**Keywords:** transcutaneous electrical acupoint stimulation (TEAS), infertility, *in vitro* fertilization-embryo transfer, systematic review, meta-analysis

## Abstract

**Background:**

Infertility is a common health problem affecting couples of childbearing age. The proposal of *in vitro* fertilization-embryo transfer (IVF-ET) solves the problem of infertility to a certain extent. However, the average success rate of IVF-ET is still low. Some studies conclude that transcutaneous electrical acupoint stimulation (TEAS) could improve pregnancy outcomes in women undergoing IVF-ET, however, there is a lack of comprehensive synthesis and evaluation of existing evidence.

**Objective:**

To conduct a systematic review and meta-analysis to assess whether TEAS is effective and safe to improve the pregnancy outcomes for women undergoing IVF-ET.

**Methods:**

Eight online databases were searched from inception to 19 November 2021. In addition, four clinical trial registries were also searched, relevant references were screened, and experts were consulted for possible eligible studies. Randomized controlled trials (RCTs) that included patients with infertility who underwent IVF and used TEAS as the main adjuvant treatment vs. non-TEAS or mock intervention controls were included. The clinical pregnancy rate (CPR) was considered the primary outcome. High-quality embryo rate (HQER), live birth rate (LBR), biochemical pregnancy rate (BPR), ongoing pregnancy rate (OPR), early miscarriage rate (EMR), birth defects rate (BDR), and adverse events related to interventions were regarded as secondary outcomes. The selection, data extraction, risk of bias assessment, and data synthesis were conducted by two independent researchers using Endnote software V.9.1 and Stata 16.0 software. The Grading of Recommendations Assessment, Development and Evaluation (GRADE) system was used to evaluate the evidence quality of each outcome.

**Results:**

There were 19 RCTs involving 5,330 participants included. The results of meta-analyses showed that TEAS can improve CPR [RR = 1.42, 95% CI (1.31, 1.54)], HQER [RR = 1.09, 95% CI (1.05, 1.14)], and BPR [RR = 1.45, 95% CI (1.22, 1.71)] of women underwent IVF-ET with low quality of evidence, and improve LBR [RR = 1.42, 95% CI (1.19, 1.69)] with moderate quality of evidence. There was no significant difference in EMR [RR = 1.08, 95% CI (0.80, 1.45)] and BDR [RR = 0.93, 95% CI (0.13, 6.54)] with very low and moderate quality of evidence, respectively. A cumulative meta-analysis showed that the effective value of TEAS vs. controls was relatively stable in 2018 [RR = 1.52, 95% CI (1.35, 1.71)]. In addition, no serious adverse events associated with TEAS were reported.

**Conclusion:**

Our findings suggest that TEAS may be an effective and safe adjuvant treatment for women undergoing IVF-ET to improve pregnancy outcomes. However, the current evidence quality is considered to be limited, and more high-quality RCTs are needed for further verification in the future.

**Systematic review registration:**

https://www.crd.york.ac.uk/PROSPERO/display_record.php?ID=CRD42021238871, identifier: CRD42021238871.

## Introduction

Infertility is a disease of the reproductive system that refers to the absence of achieving a clinical pregnancy after 1 year or more of regular unprotected sexual intercourse, which is a tough public issue worldwide (1). According to statistics, the global average incidence of infertility is about 9%, affecting 8~12% of couples of childbearing age (2). Around 10~15% of couples of childbearing age in China suffer from infertility (3). The first baby conceived *via in vitro* fertilization-embryo transfer (IVF-ET) was born in the UK in 1978. The field of assisted reproductive technology (ART) began to rapidly develop after that. As the major ART component, IVF-ET has become a common medical treatment for infertility. In Europe, more than 300,000 cycles of IVF-ET are performed every year (4) with the cost of each cycle varying from US $15,000 to US $18,000, which is a great consumption of time and finance (5, 6). However, the average success rate is low (7). In recent years, some medicine, techniques, and equipment have been developed to improve the outcomes of IVF-ET. However, the improvement of pregnancy outcomes is limited (8, 9). Some researchers are investigating the efficacy of complementary and alternative therapies for IVF-ET improvement (10).

Transcutaneous electrical acupoint stimulation (TEAS), one of the complementary and alternative therapies, is a new method of stimulating acupoints with electric current (11). The TEAS is painless, non-invasive, and convenient, which is easily accepted by patients (12). The TEAS has been recently applied in many conditions (13–17) (e.g., labor, withdrawal syndrome, breast cancer, Alzheimer's disease, hemodialysis, and lung cancer). Some studies conclude that TEAS is beneficial for women undergoing IVF-ET (18–20), which can not only increase the number of oocytes retrieved and high-quality embryos (19) but also improve the endometrium (20). TEAS has been found to work mainly by regulating the neuroendocrine system and blood flow of reproductive organs (21). Nonetheless, the efficacy of TEAS for women undergoing IVF is still controversial. Therefore, this study aims to conduct a systematic review and meta-analysis to comprehensively evaluate and synthesize all the randomized controlled trials (RCTs) of TEAS for women undergoing IVF-ET and try to provide evidence for clinical treatment and further research.

## Materials and methods

### Study registration

The study has been registered on PROSPERO (CRD42021238871) and drafted according to the Preferred Reporting Items for Systematic Review and Meta-Analysis (PRISMA) guidelines (22) and A Measure Tool to Assess Systematic Reviews-2 (AMSTAR-2) (23).

### Inclusion criteria

#### Types of studies

This study included RCTs involving patients with infertility who underwent IVF-ET and used TEAS as the main adjuvant treatment vs. IVF-ET combined with non-TEAS, or mock intervention. The languages of publication were restricted to English and Chinese.

#### Type of participants

Patients who underwent IVF-ET with or without ICSI treatment were included, whether or not failure cycles existed before. Patients with infertility due to various female factors were included. Moreover, race, age, and nationality were not restricted.

#### Types of interventions

The TEAS as the main adjuvant treatment was included.

#### Types of comparator(s)/control

Non-TEAS or mock intervention as the main adjuvant treatment was included.

Both intervention and control groups were considered to take IVF-ET as the basic treatment. The protocol of IVF-ET was not restricted, but it was restricted to the same in one study.

#### Types of outcome measures

##### Primary outcomes

The clinical pregnancy rate (CPR) was regarded as the primary outcome.

##### Secondary outcomes

Secondary outcomes included assessing the quality of the embryo, pregnancy condition, neonatal condition, and safety. For embryo outcome, a high-quality embryo rate (HQER) was considered. For pregnancy outcomes, live birth rate (LBR), biochemical pregnancy rate (BPR), ongoing pregnancy rate (OPR), and early miscarriage rate (EMR) were considered. For neonatal conditions, the birth defects rate (BDR) was considered. For safety outcomes, adverse events related to interventions were considered.

### Exclusion criteria

1. Design type was non-RCT
2. The cause of infertility was only related to the male factor
3. Percutaneous electrical stimulation in the intervention group was the left the acupoints
4. The control group was taking another genuine acupuncture therapy (e.g., manual acupuncture, electro-acupuncture, auricular acupuncture, and so on) as an adjuvant therapy
5. The data were found to be significantly falsified
6. Duplicate published data
7. The full data were not available after all efforts

### Search methods for identification of studies

#### Electronic searches

Eight databases were searched from inception to 19 November 2021: Cochrane Library, MEDLINE, EMbase, PsycINFO, CINAHL, Chinese National Knowledge Infrastructure (CNKI), Wanfang Database, and the Chongqing VIP Chinese Science and Technology Periodical Database (VIP). Only RCTs evaluating the efficacy and safety of TEAS by the aforementioned controls were included. The literature searches were constructed around medical search headings (MeSH) for TEAS, MeSH for IVF, and MeSH for RCT. In addition, appropriate adjustments were made according to the necessity of each database. The specific searching strategy of English electronic databases was listed in Supplementary Table S1.

#### Searching other resources

The following clinical trial registries were searched for relevant ongoing trials and unpublished trials: the International Clinical Trials Registry Platform (http://www.who.int/ictrp/en/), the NIH clinical registry ClinicalTrials.gov (https://www.clinicaltrials.gov/), the Australian New Zealand Clinical Trials Registry (http://www.anzctr.org.au/), and the Chinese clinical registry (http://www.chictr.org/en/). The references of all identified publications were screened. In addition, experts in the field were consulted for relevant studies.

### Data collection and analysis

#### Selection of studies

The retrieved studies were imported into Endnote software V.9.1. After removing duplicates, two researchers (ZYX and JJL) screened the studies independently based on the inclusion and exclusion criteria. The initial screening was conducted by reading the titles and abstracts to determine inclusion or exclusion. Two researchers conducted a second screening by reading the full text. The reasons for the second exclusion were recorded in detail. Two researchers cross-checked the final screened results. An agreement was reached through discussion when any dispute arose. When consensus cannot be reached through discussion, a third researcher (FRL) was involved.

#### Data extraction and management

Two researchers (HY and WHH) extracted relevant data independently from included studies. Four main domains were included in the pre-designed form: basic information (title, name of the first author, year of publication, country, source of publication, and sources of funds), method (participants, intervention, control treatment, study design, and methodology), results (outcomes and adverse events), and conclusion. Two researchers have cross-checked after data extraction. An agreement was reached through discussion when any dispute arose. If consensus cannot be reached through discussion, the third researcher (JY) was involved.

#### Assessment of risk of bias of included studies

Two researchers (GXX and ZHY) used the Cochrane Collaboration's tool ROB2.0 (24) to evaluate the risk of bias for the included studies independently from the following five domains: bias arising from the randomization process, bias due to deviations from intended interventions, bias due to missing outcome data, bias in outcome measurement, and bias in the selection of the reported result. If all domains were marked *low risk*, overall bias was regarded as *low risk of bias*. If one domain was marked *some concern*, overall bias was regarded as *some concerns*. If one domain was marked *high risk* or several domains were marked *some concern* that could influence the robustness of the study, overall bias was regarded as *high risk of bias*. If the information was missing that affected the assessment of this study, the authors were contacted. The two researchers have cross-checked after completing the evaluation. An agreement was reached through discussion when any dispute arose. If consensus cannot be reached through discussion, the third researcher (JY) was involved.

#### Data analysis

Stata 16.0 software was utilized to synthesize and analyze the data statistically. A risk ratio (RR) with a 95% confidence interval (CI) was chosen to analyze the outcome of dichotomous data. Statistical heterogeneity was investigated by conducting chi-squared tests in the forest plot, and significance was considered if the *P*-value was <0.05 (25). In addition, the statistical heterogeneity in the meta-analysis was evaluated by calculating the *I*^2^ value. According to the Cochrane Handbook (25), the *I*^2^ value was suggested to be classified in the following four degrees: 0–40% (no heterogeneity), 30–60% (moderate heterogeneity), 50–90% (substantial heterogeneity), and 75–100% (considerable heterogeneity). If *I*^2^ < 40%, the fixed effects model was chosen. If 40% ≤ *I*^2^ < 75%, the random effects model was chosen. When statistical heterogeneity was significant, subgroup analysis, or sensitivity analysis, or only descriptive analysis was conducted. When heterogeneity was acceptable and the number of included trials was >15 (26), cumulative meta-analysis was performed complementally. If the number of included trials were >10, the funnel plot and Egger's test were conducted to examine reporting bias. Besides, subgroup analysis was conducted according to different clinical characteristics and methodological features.

### Evidence quality evaluation

By using the Grading of Recommendations Assessment, Development, and Evaluation (GRADE) system (27), the evidence quality of each outcome was independently evaluated by two researchers (SYY and XYZ). According to GRADE rating standards, the evidence quality was rated as *very low, low, moderate*, or *high*. The quality of evidence was mainly assessed in terms of risk of bias, inconsistency, indirectness, imprecision, publication bias, large effect, dose–response, and all plausible confounding factors (27, 28). Two researchers have cross-checked after completing the evaluation. An agreement was reached through discussion if any dispute arose. If consensus cannot be reached through discussion, a third researcher (FRL) was involved.

## Results

### Search results

**Figure 1:**
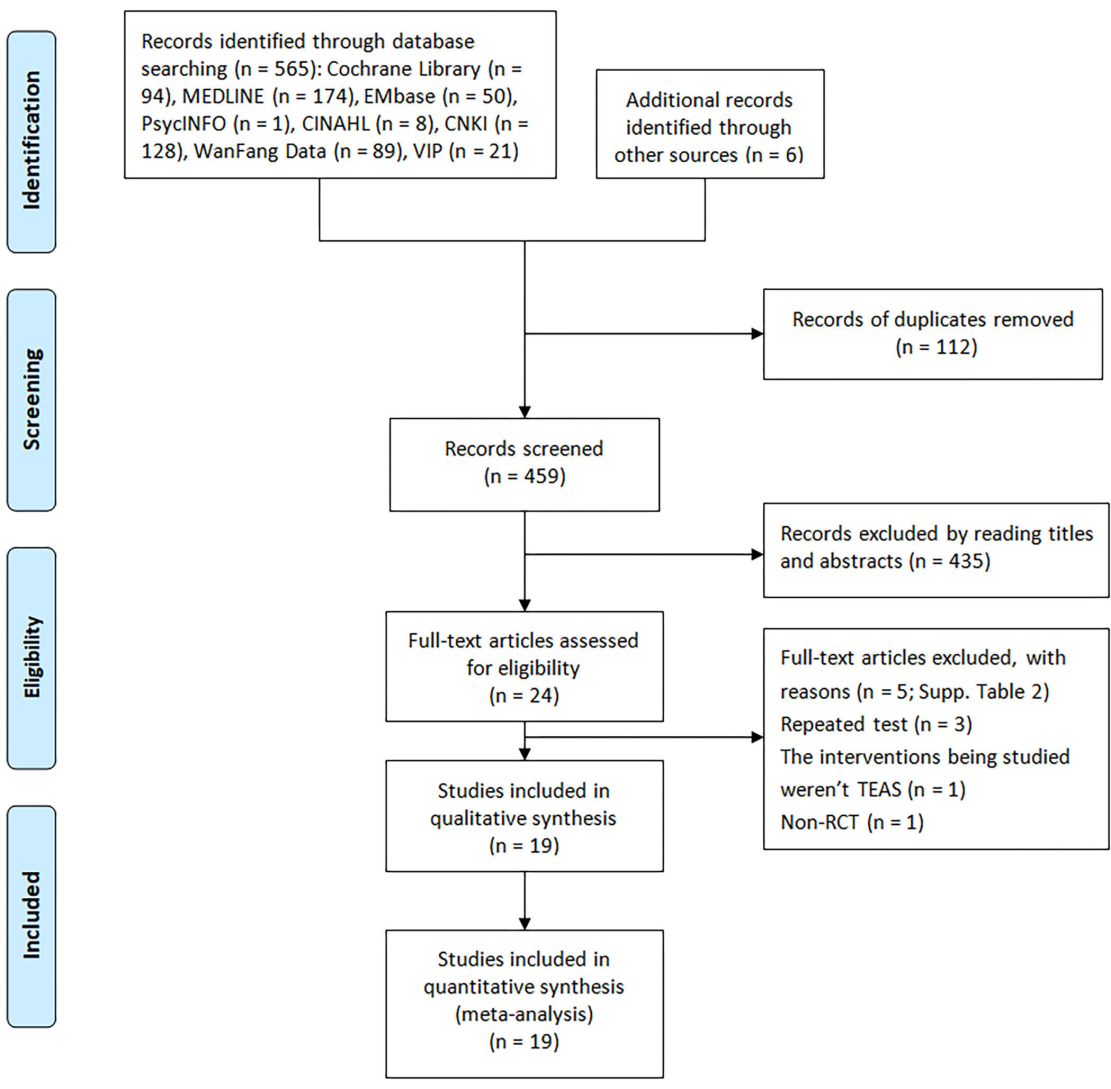
Process and results of studies selection of RCTs of TEAS for women undergoing IVF.

A total of 571 relevant studies were retrieved, including 565 from electronic databases, 5 from clinical trial registry platforms, and 1 from expert consultation. A total of 112 duplicated studies were removed by Endnote software V.9.1, and 435 studies were removed after reading titles and abstracts. A total of 5 studies were excluded after reading the full text. For duplicate data, we retained the one with higher quality and more comprehensive information. Finally, 19 RCTs (18–20, 29–43) were included. The specific study screening process and results are shown in Figure 1, and the specific exclusion information of the secondary screening is shown in Supplementary Table S2.

### Characteristics of included studies

All 19 included studies were RCTs from China. The published years were from 2011 to 2022. There were 6 RCTs published in English and 13 in Chinese, among which 6 were master dissertations in Chinese. A total of 5,330 patients diagnosed with infertility were included. Of all included studies, there were 14 two-arm studies (20, 30–40, 43), 3 three-arm studies (18, 19, 41), 1 four-arm study (29), and 1 five-arm study (42). A total of 5 RCTs (29, 31, 37, 40, 41) were restricted eligibility to fresh ET, 4 RCTs (20, 30, 35, 39) were restricted to frozen-thawed embryo transfer (FET), 4 RCTs (18, 33, 42) were without restriction on embryo type, and 6 RCTs did not mention it. Some of the participants in 5 RCTs (18, 30, 33, 41, 42) used ICSI, 4 RCTs (19, 20, 29, 40) excluded patients using ICSI, and 10 RCTs did not mention it. Four studies (20, 31, 35, 39) only enrolled patients with a history of IVF failure, two studies (29, 40) excluded these patients, three studies (18, 41, 42) recruited patients with or without the history, and 10 studies did not mention it. Eight trials (18, 20, 29, 30, 33, 41, 42) reported the specific times of TEAS intervention, and among them, six trials (18, 29, 30, 33, 42) set it to 1 time or 2 times. In addition, 11 studies (19, 31, 32, 34–40, 43) performed TEAS in the 2 or 3 menstrual cycles prior to IVF-ET/FET and/or during IVF-ET/FET, while they did not mention the specific times. There were 9 studies (18, 29–31, 33, 37, 41, 42) that operated TEAS on the ET day. TEAS was administered for 30 min per time in each study except for 20 min in 1 study (38). No TEAS were set as controls in 13 studies (19, 29, 30, 34–39, 41–43), and mock interventions were set as controls in 8 studies, including 1 study (32) setting mock acupuncture group and 7 (18–20, 31, 33, 40, 42) setting mock TEAS groups. In 1 study (41), a G6805-II percutaneous electric nerve stimulator was used for TEAS. In two studies (18, 39), no specific equipment was mentioned, and in the other 16 studies, Han's device was used. CPR was reported in all included studies and HQER was reported in seven studies (19, 32, 36–38, 40, 43). LBR was reported in eight studies (18, 20, 29, 31, 40–42), BPR was reported in nine studies (18, 30, 32, 33, 35, 37, 39, 42), EMR was reported in five studies (19, 33, 41, 42), BDR was reported in one study, and no study was reported on OPR. One study (29) claimed to have no adverse events, and two trials (19) documented that the incidence and severity of adverse events in the control groups were higher than that in the TEAS group. The rest did not mention adverse events. Ten studies reported funding and six studies declared no conflicts of interest. The detailed characteristics of all included studies were shown in Supplementary Table S4.

### Risk of bias assessment

The overall risk of bias of two studies (33, 40) was considered high risk, five studies (20, 29, 31, 41) were considered low risk, and the remaining 12 studies (18, 19, 30–32, 34–37, 39, 42, 43) were considered some concerns. A total of 14 studies (18, 19, 30–37, 39, 40, 42, 43) in domain 1 (randomization process) were considered some concerns, and the rest were considered low risk. Two studies (33, 40) in domain 2 (deviations from the intended interventions) were considered some concerns, and the rest were considered low risk. As for domain 3 (missing outcome data), domain 4 (measurement of the outcome), and domain 5 (selection of the reported result), all included RCTs were considered low risk. The summary of the assessment of the risk of bias for each included study by ROB 2.0 is shown in Table 1, and the details of the assessment are shown in Supplementary Table S3.

**Table 1:** Assessment of risk of bias of included studies using the tool RoB 2.0.

**References**	**Randomization process**	**Deviations from the intended interventions**	**Missing outcome data**	**Measurement of the outcome**	**Selection of the reported result**	**Overall risk-of-bias judgment**
Qu et al. (29)	Low risk	Low risk	Low risk	Low risk	Low risk	Low risk
Shuai et al. (20)	Low risk	Low risk	Low risk	Low risk	Low risk	Low risk
Zhang et al. (18)	Some concerns	Low risk	Low risk	Low risk	Low risk	Some concerns
Zheng et al. (19)	Some concerns	Low risk	Low risk	Low risk	Low risk	Some concerns
Feng (30)	Some concerns	Low risk	Low risk	Low risk	Low risk	Some concerns
Shuai et al. (31)	Some concerns	Low risk	Low risk	Low risk	Low risk	Some concerns
Zhao et al. (32)	Some concerns	Low risk	Low risk	Low risk	Low risk	Some concerns
Li (33)	Some concerns	Some concerns	Low risk	Low risk	Low risk	High risk
Zhu (34)	Some concerns	Low risk	Low risk	Low risk	Low risk	Some concerns
Li et al. (35)	Some concerns	Low risk	Low risk	Low risk	Low risk	Some concerns
Chen (36)	Some concerns	Low risk	Low risk	Low risk	Low risk	Some concerns
Dong et al. (37)	Some concerns	Low risk	Low risk	Low risk	Low risk	Some concerns
Shuai and Yang (38)	Low risk	Low risk	Low risk	Low risk	Low risk	Low risk
Xu et al. (39)	Some concerns	Low risk	Low risk	Low risk	Low risk	Some concerns
Fang et al. (40)	Some concerns	Some concerns	Low risk	Low risk	Low risk	High risk
Zhang and Zhong (41)	Low risk	Low risk	Low risk	Low risk	Low risk	Low risk
Feng (42)	Some concerns	Low risk	Low risk	Low risk	Low risk	Some concerns
Mi (43)	Some concerns	Low risk	Low risk	Low risk	Low risk	Some concerns
Feng, under review	Low risk	Low risk	Low risk	Low risk	Low risk	Low risk

### Effects of intervention

The comparison results and GRADE analyses are summarized and shown in Table 2. Based on different outcomes, we synthesized and analyzed data in the TEAS group vs. the control group. According to different types of control groups and different degrees of risk of bias, we conducted subgroup analyses.

**Table 2:** Quality of evidence included RCTs by GRADE.

**Outcome**	**Included RCTs (participants)**	**Relative effect (95% CI)**	**Risk of bias**	**Inconsistency**	**Indirectness**	**Imprecision**	**Publication bias**	**Quality of evidence**
CPR	19 (3,784)	RR 1.42 (1.31–1.54)	−1^①^	0	0	0	−1^③^	Low
HQER	7 (753)	RR 1.09 (1.05–1.14)	−1^①^	0	0	0	−1^④^	Low
LBR	7 (2,174)	RR 1.47 (1.28–1.67)	0	0	0	0	−1^④^	Moderate
BPR	8 (1,191)	RR 1.57 (1.37–1.80)	−1^①^	0	0	0	−1^④^	Low
EMR	4 (636)	RR 0.88 (0.61–1.26)	−1^①^	0	0	−1^②^	−1^④^	Very low
BDR	1 (731)	RR 0.93 (0.13–6.54)	0	0	0	0	−1^④^	Moderate

CPR, clinical pregnancy rate; HQER, high-quality embryo rate; LBR, live birth rate; BPR, biochemical pregnancy rate; EMR, early miscarriage rate; BDR, birth defects rate.

① Most information is from the studies whose risk of bias with some concerns, and there are major limitations.

② The sample is insufficient.

③ Asymmetric funnel plots suggest that there may be a publication bias.

④ Few studies are included, and there may be a large publication bias.

#### Clinical pregnancy rate

**Figure 2:**
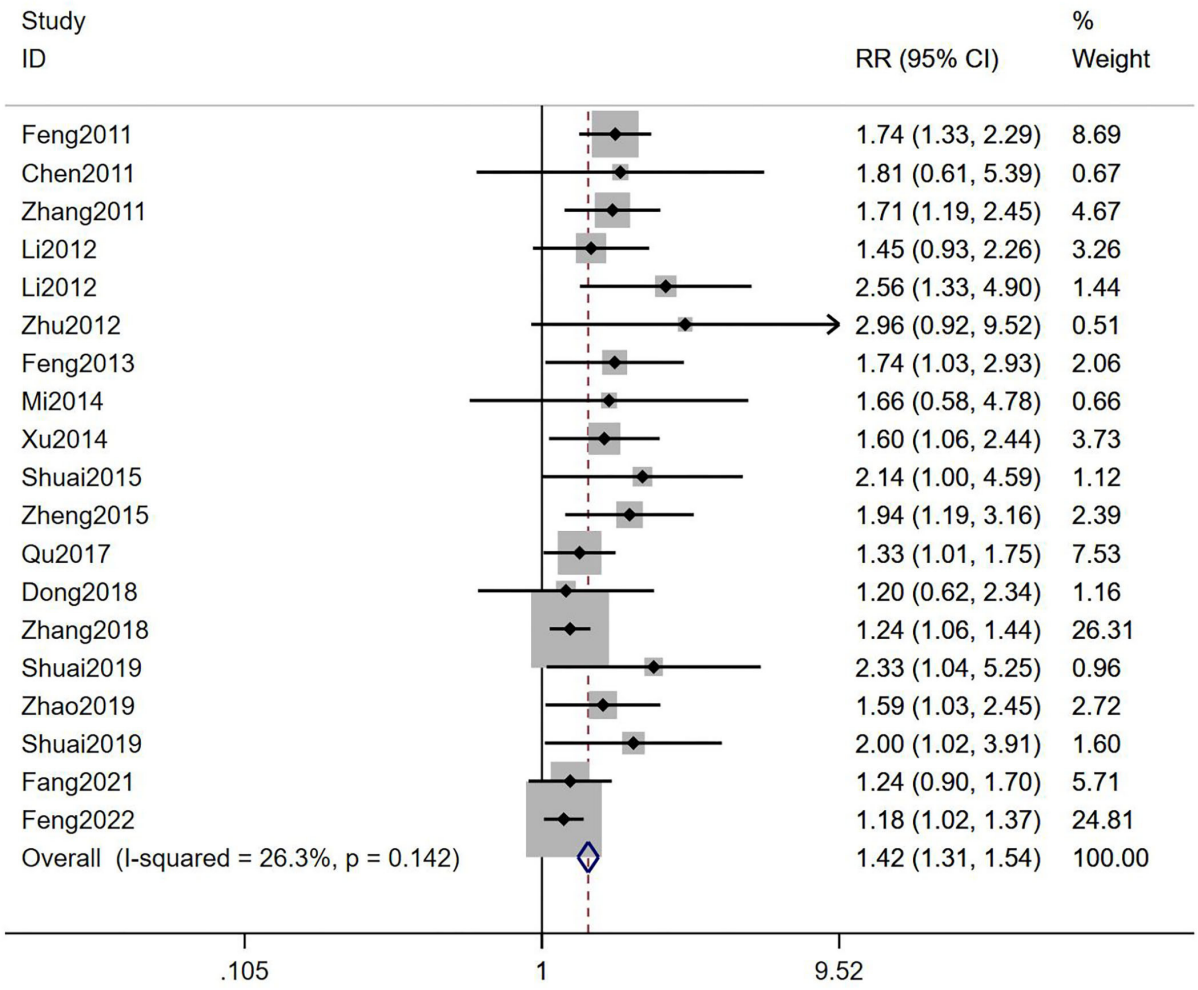
Meta-analysis of the effects of TEAS compared to controls in CPR.

It was suggested that compared with the control groups, TEAS could improve CPR of infertile women who accepted IVF-ET [RR = 1.42, 95% CI (1.31, 1.54), *I*^2^ = 26.3%; Figure 2], although the quality of evidence was low (Table 2). The results showed that TEAS-treated IVF-ET patients had statistical difference in CPR compared with mock intervention or no TEAS control [RR = 1.66, 95% CI (1.42, 1.95), *I*^2^ = 0%; RR = 1.34, 95% CI (1.22, 1.47), *I*^2^ = 17.9%; Table 3]. Besides, whether in low risk group, some concerns group, or high risk group, TEAS showed an advantage in improving CPR [RR = 1.26, 95% CI (1.14, 1.39), *I*^2^ = 13.6%; RR = 1.78, 95% CI (1.54, 2.06), *I*^2^ = 0%; RR = 1.32, 95% CI (1.02, 1.70), *I*^2^ = 0%; Table 3].

**Table 3:** Summary of overall meta-analyses and subgroup analyses.

**Factor**	**Outcome or subgroup**	**No. of studies**	**Effect (95% CI)**	***I* ^2^**
	**CPR**
Type of control group	Mock intervention	9	1.66 (1.42,1.95)	0%
	No TEAS	12	1.34 (1.22,1.47)	17.9%
Risk of bias	Low risk	5	1.26 (1.14,1.39)	13.6%
	Some concerns	12	1.78 (1.54,2.06)	0%
	High risk	2	1.32 (1.02,1.70)	0%
	Total	19	1.42 (1.31,1.54)	26.3%
	**HQER**
Type of control group	Mock intervention	5	1.10 (1.04,1.16)	0%
	No TEAS	4	1.08 (1.00,1.17)	31.0%
Risk of bias	Some concerns	6	1.10 (1.04,1.16)	30.6%
	High risk	1	1.08 (1.00,1.16)	/
	Total	7	1.09 (1.05,1.14)	20.2%
	**LBR**
Type of control group	Mock intervention	5	1.73 (1.37,2.17)	0%
	No TEAS	4	1.23 (1.09,1.39)	34.3%
Risk of bias	Low risk	4	1.22 (1.07,1.38)	28.9%
	Some concerns	3	1.86 (1.46,2.36)	0%
	High risk	1	1.20 (0.78,1.87)	/
	Total	8	1.42 (1.19,1.69)	52.0%
	**BPR**
Type of control group	Mock intervention	4	1.63 (1.35,1.96)	0%
	No TEAS	6	1.35 (1.10,1.67)	49%
Risk of bias	Low risk	1	1.08 (0.94,1.25)	/
	Some concerns	7	1.57 (1.36,1.82)	0%
	High risk	1	1.45 (0.97,2.18)	/
	Total	9	1.45 (1.22,1.71)	53.2%
	**EMR**
Type of control group	Mock intervention	3	0.60 (0.24,1.51)	0%
	No TEAS	4	1.16 (0.85,1.58)	2.2%
Risk of bias	Low risk	2	1.22 (0.88,1.68)	46.7%
	Some concerns	2	0.65 (0.29,1.47)	0%
	High risk	1	0.44 (0.08,2.43)	/
	Total	5	1.08 (0.80,1.45)	17.3%
	**BDR**
	Total	1	0.93 (0.13,6.54)	/

No., number; CPR, clinical pregnancy rate; HQER, high-quality embryo rate; LBR, live birth rate; BPR, biochemical pregnancy rate; EMR, early miscarriage rate; BDR, birth defects rate; TEAS, transcutaneous electrical acupoint stimulation.

**Figure 3:**
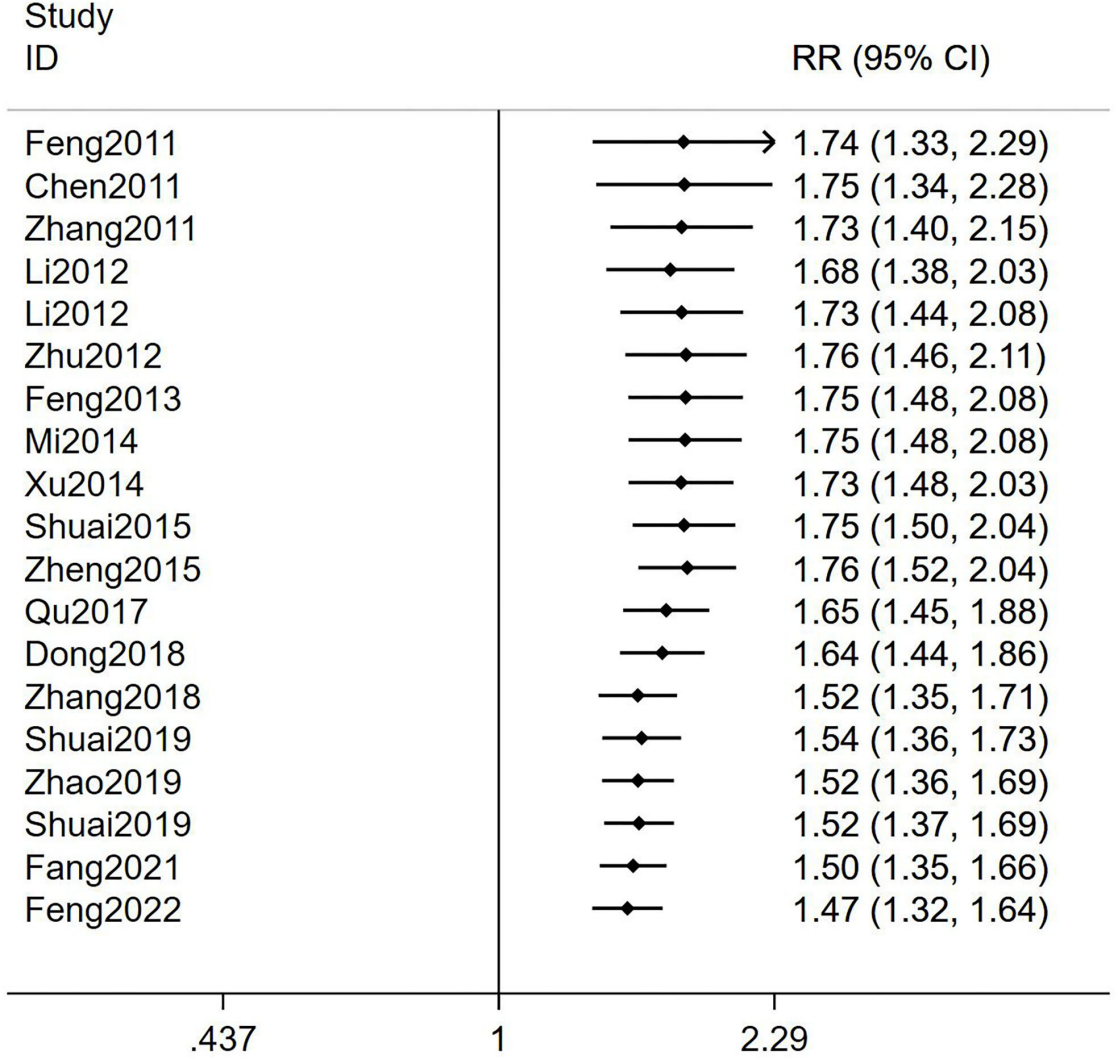
Cumulative meta-analysis of the effects of TEAS compared to controls in CPR (according to years of publication).

In addition, a cumulative meta-analysis according to years of publication showed that TEAS was shown to be statistically different in CPR for infertile women undergoing IVF-ET as compared to controls in 2011 [RR = 1.74, 95% CI (1.33, 2.29)]. At the same time, through longitudinal comparison, we found that RR values tended to be stable after 2018 [RR = 1.52, 95% CI (1.35, 1.71)], and the 95% CI gradually narrowed, suggesting that the accuracy gradually increased over time. The final RR value stayed at 1.47 in 2022, with 95% CI (1.32, 1.64) suggesting that the TEAS group had higher CPR than the control group and tended to stabilize over time (Figure 3).

#### High-quality embryo rate

**Figure 4:**
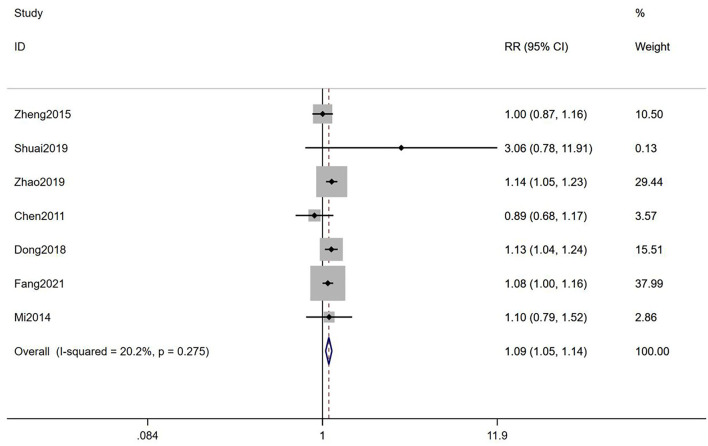
Meta-analysis of the effects of TEAS compared to controls in HQER.

The result showed that TEAS could improve HQER [RR = 1.09, 95% CI (1.05, 1.14), *I*^2^ = 20.2%; Figure 4] with low-quality evidence (Table 2). When compared with mock intervention, it showed that TEAS treated IVF-ET patients had statistical difference in HQER [RR = 1.10, 95% CI (1.04, 1.16), *I*^2^ = 0%; Table 3], while there was no significant statistical differences when compared with no TEAS [RR = 1.08, 95% CI (1.00, 1.17), *I*^2^ = 31.0%; Table 3]. In some concerns group, TEAS showed advantages in improving HQER [RR = 1.10, 95% CI (1.04, 1.16), *I*^2^ = 30.6%; Table 3]. However, there was no significant statistical differences in the high risk group [RR = 1.08, 95% CI (1.00, 1.16); Table 3].

#### Live birth rate

**Figure 5:**
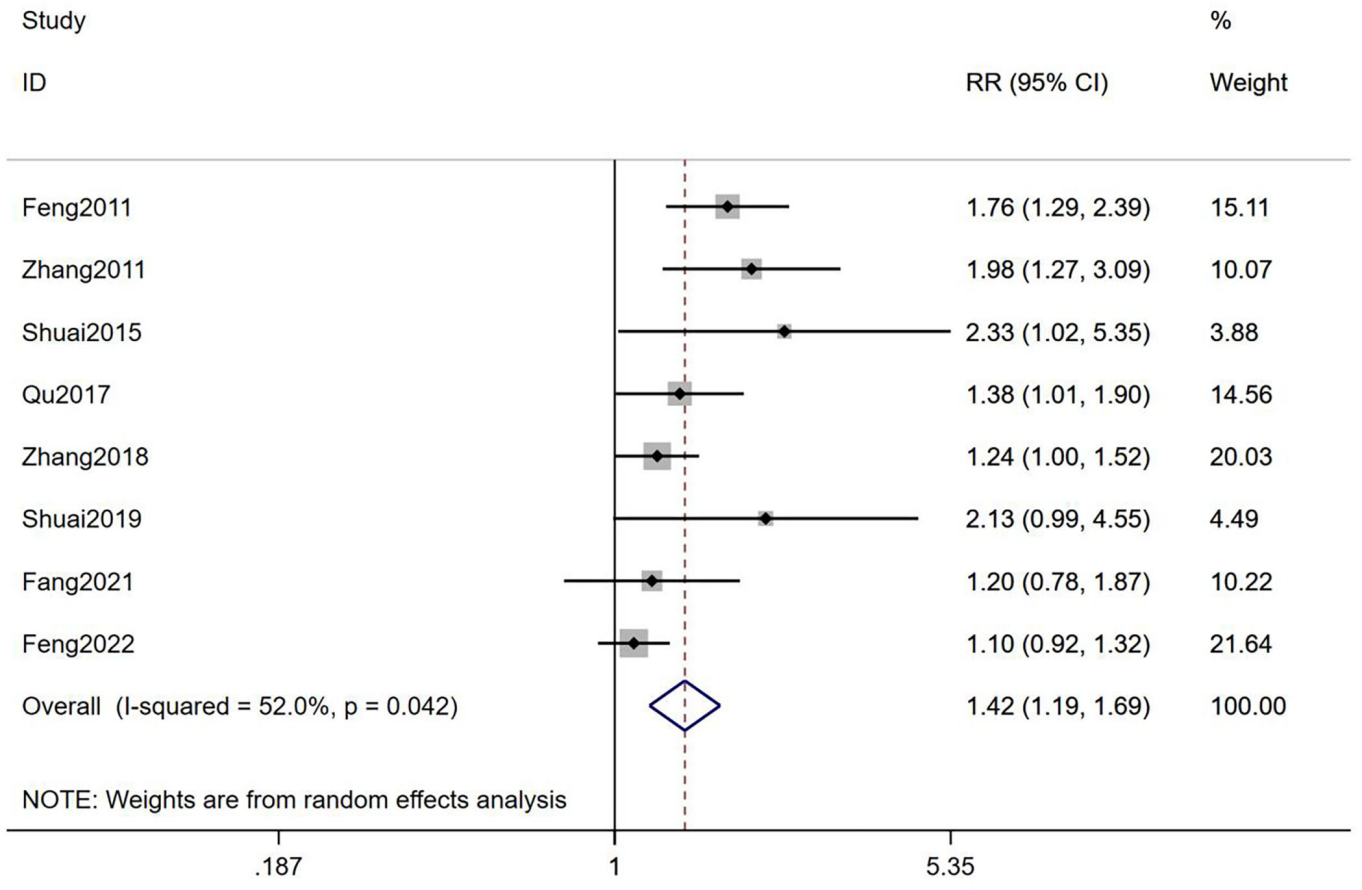
Meta-analyses of the effects of TEAS compared to controls in LBR.

It was suggested that TEAS could improve LBR of infertile women who accepted IVF-ET compared with the control groups [RR = 1.42, 95% CI (1.19, 1.69), *I*^2^ = 52.0%; Figure 5], with a moderate-quality evidence (Table 2). According to the results of subgroup analyses, it showed that TEAS had statistical difference when compared with both mock intervention and no TEAS [RR = 1.73, 95% CI (1.37, 2.17), *I*^2^ = 0%; RR = 1.23, 95% CI (1.09, 1.39), *I*^2^ = 34.3%; Table 3]. In addition, TEAS showed statistical differences in improving LBR in both the low risk group and the some concerns group [RR = 1.22, 95% CI (1.07, 1.38), *I*^2^ = 28.9%; RR = 1.86, 95% CI (1.46, 2.36), *I*^2^ = 0%], but not in the high risk group [RR = 1.20, 95% CI (0.78, 1.87); Table 3].

#### Biochemical pregnancy rate

**Figure 6:**
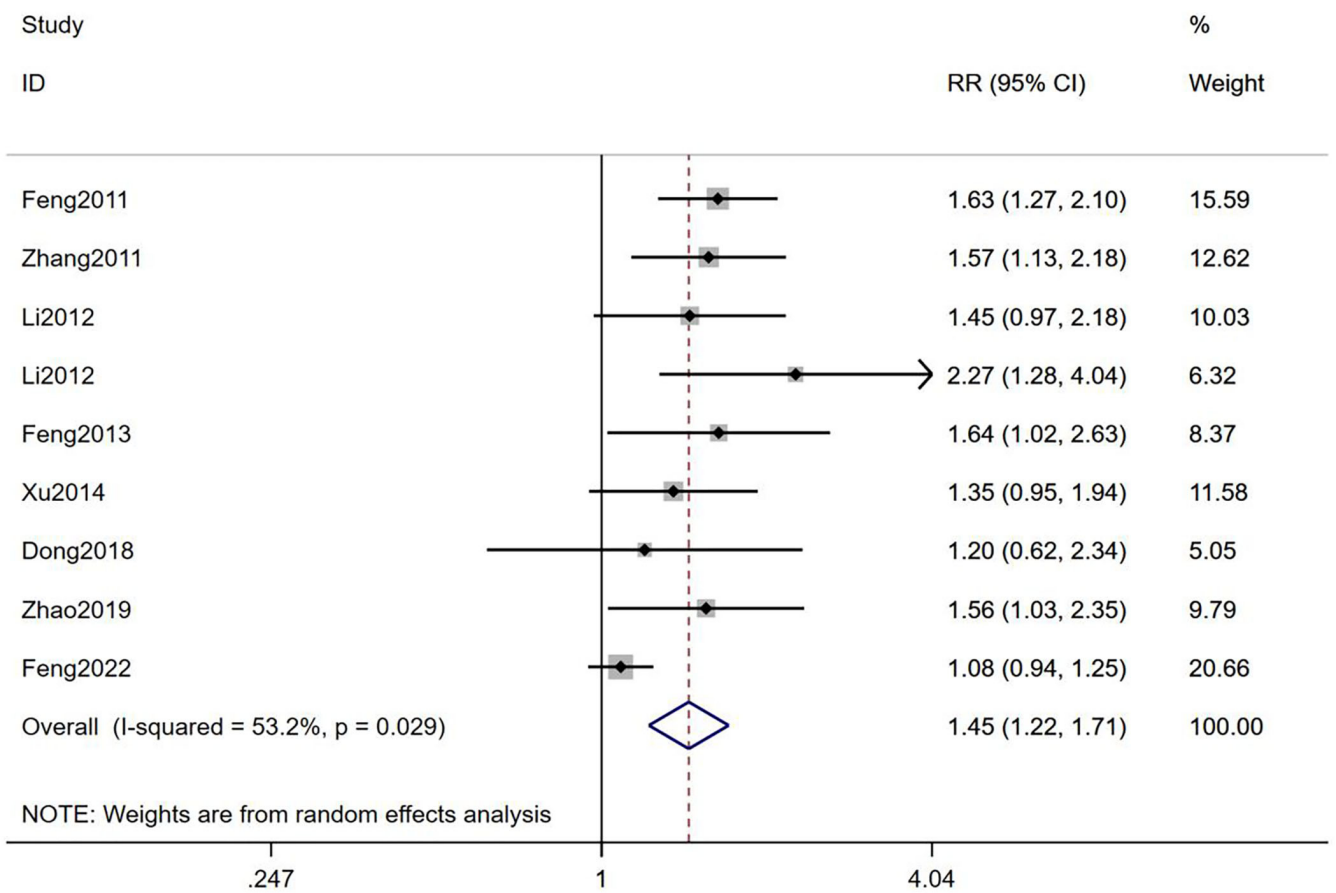
Meta-analyses of the effects of TEAS compared to controls in BPR.

It was suggested that TEAS could improve BPR of infertile women who accepted IVF-ET compared with the control groups [RR = 1.45, 95% CI (1.22, 1.71), *I*^2^ = 53.2%; Figure 6], with a low-quality evidence (Table 2). The result of subgroup analyses showed that TEAS had statistical difference in improving BPR, compared with mock intervention or no TEAS [RR = 1.63, 95% CI (1.35, 1.96), *I*^2^ = 0%; RR = 1.35, 95% CI (1.10, 1.67), *I*^2^ = 49%; Figure 6]. Besides, whether in low risk group, some concerns group, or high risk group, TEAS showed an advantage [RR = 1.08, 95% CI (0.94, 1.25); RR = 1.57, 95% CI (1.36, 1.82), *I*^2^ = 0%; RR = 1.45, 95% CI (0.97, 2.18), *I*^2^ = 0%; Table 3].

#### Early miscarriage rate

**Figure 7:**
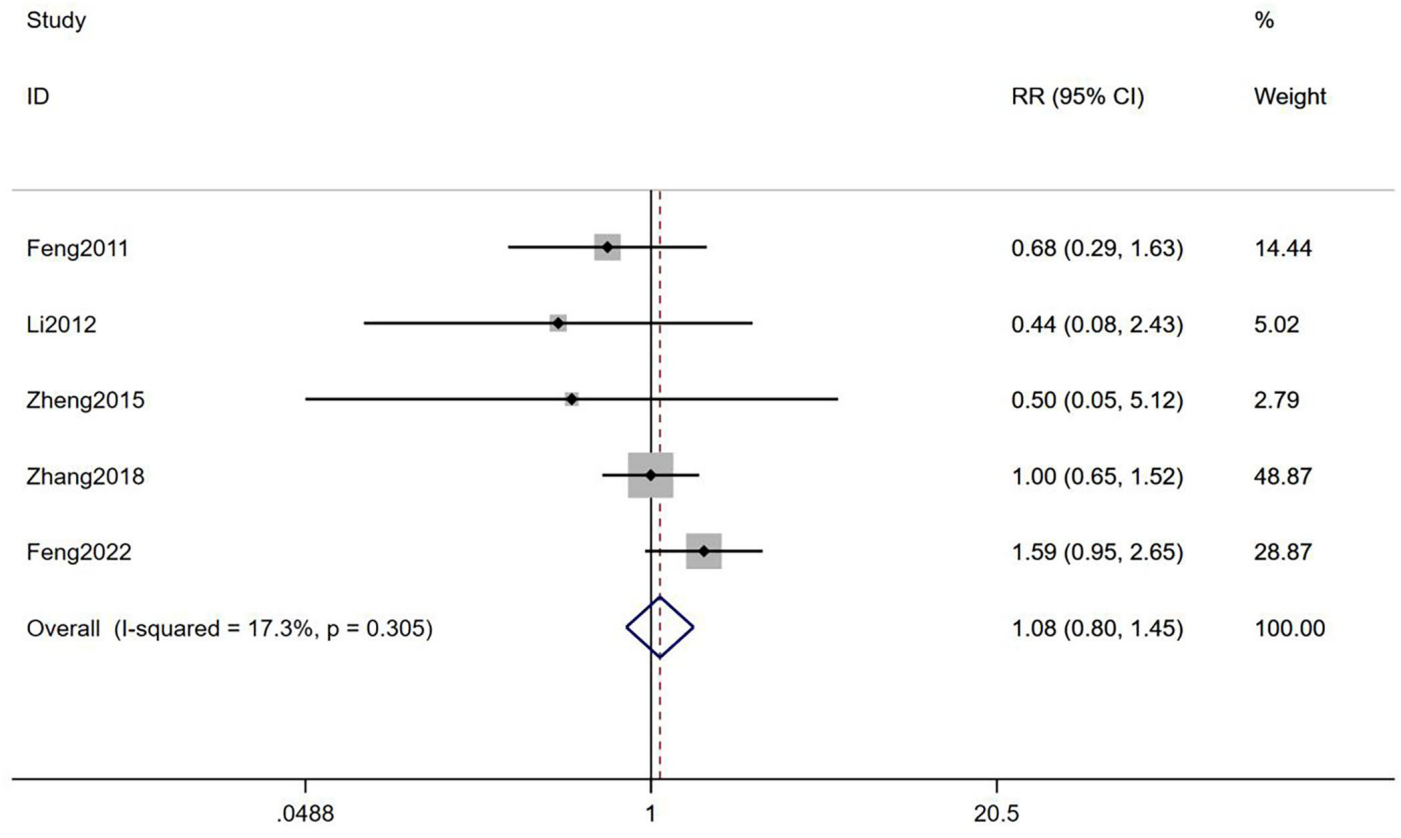
Meta-analyses of the effects of TEAS compared to controls in EMR.

It was suggested that there was no statistical difference between TEAS and controls in EMR [RR = 1.08, 95% CI (0.80, 1.45), *I*^2^ = 17.3%; Figure 7] with a very low-quality evidence (Table 2). When compared with mock intervention or no TEAS, there was no statistical difference in EMR [RR = 0.60, 95% CI (0.24, 1.51), *I*^2^ = 0%; RR = 1.16, 95% CI (0.85, 1.58), *I*^2^ = 2.2%; Table 3]. Besides, it showed no statistical differences in low risk group, some concerns group, and high risk group [RR = 1.08, 95% CI (0.94, 1.25); RR = 1.57, 95% CI (1.36, 1.82), *I*^2^ = 0%; RR = 1.45, 95% CI (0.97, 2.18); Table 3].

#### Birth defects rate

There was only 1 study that reported BDR, and it showed no statistical difference between the TEAS group and control group [RR = 0.93, 95% CI (0.13, 6.54); Table 3].

### Safety of intervention

Adverse events were reported in two studies (19): 1 study reported 2 cases of mild allergy in the TEAS group, 1 case of mild allergy in the mock TEAS group, and 3 cases of mild liver function abnormalities, 7 cases of dizziness, and 3 cases of fatigue in the non-TEAS group. The other one reported TEAS had no detrimental adverse events profile compared with the control group. Besides, 1 study (29) reported that there was no adverse event. None of the other studies mentioned the safety of the intervention.

### Reporting bias

**Figure 8:**
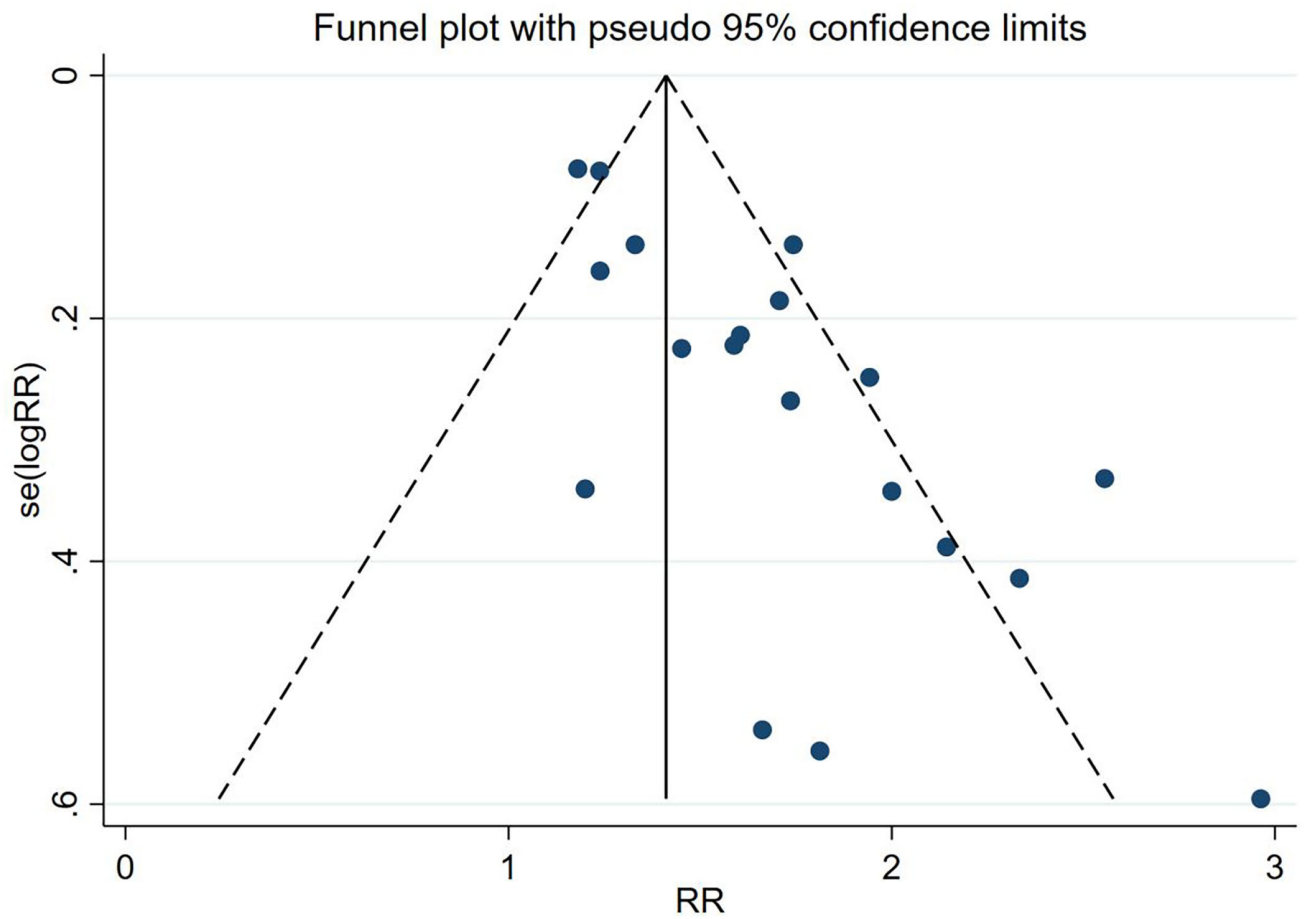
Funnel plots of included studies on CPR.

**Figure 9:**
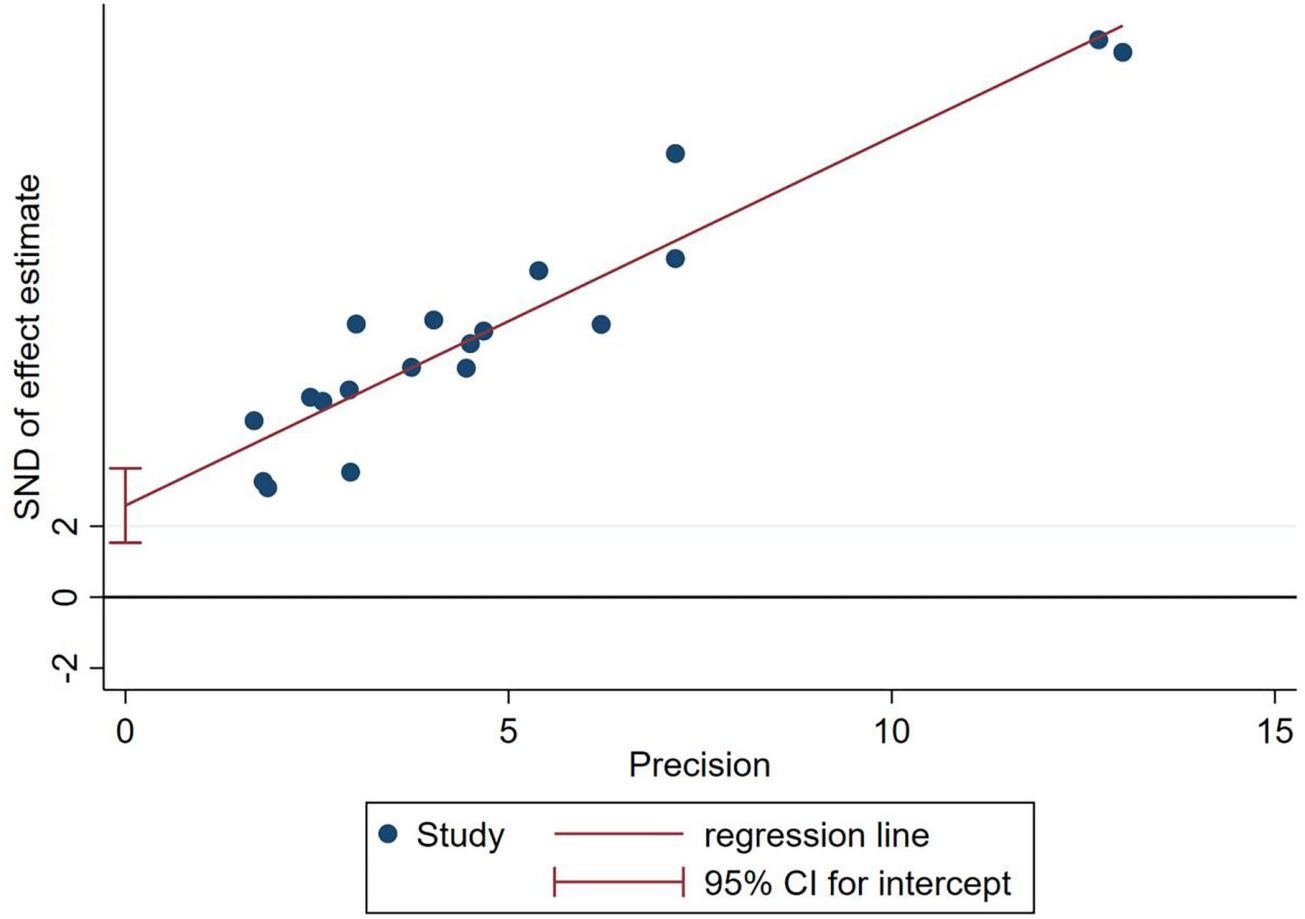
Egger's test of included studies on CPR.

Due to the insufficient number of studies reporting the outcomes, except for CPR, only one funnel plot and Egger's test were conducted. Asymmetrical funnel plots and Egger's test (*P* < 0.001) suggest the possibility of publication bias (Figures 8, 9).

### Quality of evidence

The GRADE system was applied to assess the quality of the evidence of included RCTs for each outcome, and the details are presented in Table 2. There were a total of 6 synthesized outcomes (CPR, HQER, LBR, BPR, EMR, and BDR) that were assessed. The results showed that there were 2 outcomes with moderate-quality evidence, 3 with low-quality evidence, 1 with very low-quality evidence, and no evidence with high quality. Most RCTs considered some concerns about the overall risk of bias due to methodological flaws in the randomization process, which did not report the specific method of randomization and whether allocation concealment was performed, which lead to a downgrade. In addition, the possibility of publication bias was also one of the important reasons leading to degraded evidence quality (Supplementary Table S3).

## Discussion

Considering the importance of evidence-based guidance (44, 45), we conducted a systematic review and meta-analysis of 19 RCTs with 5,330 participants to assess whether TEAS improves pregnancy outcomes in infertile women undergoing IVF-ET and its safety. In our study, TEAS and controls were found to show statistical differences in CPR, HQER, LBR, and BPR, whereas it showed no statistical difference in EMR and BDR. In addition, three studies looked at the safety of the intervention and did not report any serious adverse events related to TEAS. As far as the evidence was concerned, TEAS may be effective and safe for improving pregnancy outcomes in women who underwent IVF-ET.

The TEAS is a non-invasive treatment developed by traditional acupuncture (46), which is easy to learn and quantify, and the stimulation is continuous and quantitative. It is a potential and effective option for patients who are unwilling to undergo invasive and painful acupuncture treatments. The TEAS is reported to increase the number and quality of ovum (19, 32, 34, 36, 38), and improve the fertilization rate and high-quality embryo rate (37, 40). Besides, it can improve endometrial receptivity (20, 30, 35, 39, 42, 43) and relieve anxiety (33), so as to improve the pregnancy outcome. In addition, TEAS also reduces the amount and duration of the use of gonadotropin, thereby reducing the financial burden on patients (19, 37).

No matter whether the session is 1 time or over 3 menstrual cycles, they all showed obvious benefits for improving pregnancy outcomes. The more times of intervention, the more benefits were shown (18, 41, 42). However, as there were fewer RCTs that compared doses of TEAS currently, the optimal intervention dose could not be determined. In addition, there was no standardized acupoints prescription. Some studies used one acupoints prescription throughout the whole process, while some studies used different acupoints prescriptions according to different stages. In general, there are 6 main stages of TEAS treatment in the included studies: before IVF, during IVF, before FET, during FET, before ET, and after ET. At the 4 stages (before IVF, during IVF, before FET, and during FET), the selected acupoints were mainly for tonifying the kidney and regulating the vital and the conception vessel, including ST25 (Tianshu), RN4 (Guanyuan), RN3 (Zhongji), Ex-CA1 (Zigong), BL23 (Shenshu), DU3 (Yaoyangguan), DU4 (Mingmen), and SP6 (Sanyinjiao). Before ET, RN12 (Zhongwan), RN4 (Guanyuan), ST29 (Guilai), EX-CA1 (Zigong), SP10 (Xuehai), SP8 (Diji), and PC6 (Neiguan) were chosen to increase blood flow and regulate the mind. After ET, the nourishing acupoints were selected, mainly including ST36 (Zusanli), KI3 (Taixi), BL23 (Shenshu), RN4 (Guanyuan), and RN12 (Zhongwan).

The Cochrane Collaboration's guidelines proposed that publication bias should be thought of as one of the possible explanations of asymmetric funnel plots rather than all (47). In our study, we contacted authors who had registered related protocols without results published but did not get data that could be included in our study. Asymmetric funnel plot may also be due to other types of reporting bias (e.g., multiple repeated publication bias, such as negative results reported only as abstracts in conferences, and language bias) or clinical heterogeneity between studies (e.g., the different incidence of positive events in control groups) (48). After exclusion one by one, we thought that in addition to publication bias, language bias and clinical heterogeneity had some possibilities to be the reasons for the asymmetric funnel plot in our study. Due to limited conditions, we only included studies published in English and Chinese. Different languages have different tendencies to publish research, which should be taken into account. In addition, we did not limit the causes of infertility, resulting in considerable differences in the basic pregnancy outcomes of the control groups between different studies.

Most of the included studies did not mention specific randomization methods and allocation concealment, and the possibility of publication bias collectively affected the quality of evidence. Considering the objectivity of the outcomes in our study and since TEAS was only used as an adjuvant treatment in IVF-ET, which was not performed simultaneously with ET, whether to use a blind method did not have a great impact on the outcomes (49), and whether to use mock intervention did not affect the operation of blinding for doctors performing ET (50, 51).

Our study has the following limitations worthy of careful consideration: first, the studies not in the English databases and Chinese databases were not searched, which may have negative results that can affect our existing results. Second, all the included studies were conducted in China, and the promotion of the conclusions may have some limitations. Third, except LBR and BDR, the quality of evidence of other outcomes was low or very low. Future research may have a significant impact on the existing evidence and may change the evaluation results. Fourth, we originally planned to perform additional subgroup analyses based on participant age, different causes of infertility, and TEAS intervention dose. However, in the end, because most of the subjects included in the RCTs were of mixed age and mixed cause of infertility, the subgroup analyses were difficult to conduct.

In general, based on the current evidence, TEAS may be an effective and safe adjuvant treatment for women undergoing IVF-ET to improve pregnancy outcomes, and easy to operate in the hospital by medical workers or at home by patients themselves. However, considering its evidence quality, more high-quality RCTs are needed for further verification in the future (52). In addition, future research needs to study the optimal scheme of TEAS, including intervention timing, intervention dose, frequency, and acupoints prescription, so as to maximize the curative effect.

## Conclusion

Our findings suggest that TEAS may be an effective and safe adjuvant treatment for women undergoing IVF-ET to improve pregnancy outcomes. However, the current evidence quality is considered to be limited, and more high-quality RCTs are needed for further verification in the future.

## Data availability statement

The original contributions presented in the study are included in the article/Supplementary material, further inquiries can be directed to the corresponding author/s.

## Author contributions

Conception and design: HY. Administrative support: W-hH, JY, and F-rL. Provision of study materials or patients: HY, G-xX, J-jL, and Z-yX. Collection and assembly of data: HY, W-hH, J-jL, G-xX, Z-yX, S-yY, and JY. Data analysis and interpretation: HY, G-xX, Z-hY, X-yZ, S-yY, and JY. Manuscript writing and final approval of manuscript: All authors.

## Funding

This study was supported by grants from the National Natural Science Foundation of China (82174517).

## Conflict of interest

The authors declare that the research was conducted in the absence of any commercial or financial relationships that could be construed as a potential conflict of interest.

## Publisher's note

All claims expressed in this article are solely those of the authors and do not necessarily represent those of their affiliated organizations, or those of the publisher, the editors and the reviewers. Any product that may be evaluated in this article, or claim that may be made by its manufacturer, is not guaranteed or endorsed by the publisher.
