# Supplementary material for: Transcutaneous electrical acupoint stimulation for pregnancy outcomes in women undergoing in vitro fertilization-embryo transfer: A systematic review and meta-analysis
Source: Front Public Health. 2022 Aug 11;10:892973. doi: 10.3389/fpubh.2022.892973 (PMC9403762; doi:10.3389/fpubh.2022.892973)
Supplement: Supplementary file 1 [file Table_1.pdf]

Supplementary Table 1 The Search Strategy of English Electronic Databases.

Medline (Ovid)

1 "nerve stimulat*".ab,ti.
2 electroacupuncture.ab,ti.
3 neuro modulation.ab,ti.
4 electro acupuncture.ab,ti.
5 neuromodulation.ab,ti.
6 "trans-abdominal stimulat*".ab,ti.
7 "transcutaneous electr* stimulat*".ab,ti.
8 "interferential electr* stimulat*".ab,ti.
9 medtronic.ab,ti.
10 or/1-9
11 exp embryo transfer/ or exp fertilization in vitro/ or exp sperm injections, intracytoplasmic/ or exp zygote intrafallopian transfer/
12 (in Vitro adj2 fertili$).tw.
13 (ivf or icsi or ZIFT).tw.
14 (intracytoplas$ adj2 sperm).tw.
15 zygote intrafallopian transfer$.tw.
16 (embryo transfer$ or ET).tw.
17 invitro fertili$.tw.
18 or/11-17
19 randomized controlled trial.pt.
20 controlled clinical trial.pt.
21 randomized.ab.
22 placebo.tw.
23 clinical trials as topic.sh.
24 randomly.ab.
25 trial.ti.
26 (crossover or cross-over or cross over).tw.
27 or/19-26
28 exp animals/ not humans.sh.
29 27 not 28
30 10 and 18 and 29

Cochrane Library

#1 nerve stimulat*:ti,ab,kw
#2 electro-acupuncture:ti,ab,kw
#3 electroacupuncture:ti,ab,kw
#4 neuro-modulation:ti,ab,kw
#5 neuromodulation:ti,ab,kw
#6 trans-abdominal stimulat*:ti,ab,kw
#7 sacral nerve stimulat*:ti,ab,kw
#8 transcutaneous electr* stimulat*:ti,ab,kw

#9 interferential electr* stimulat*:ti,ab,kw
#10 "medtronic":ti,ab,kw
#11 "SXDZ-100":ti,ab,kw
#12 "SDZ-II":ti,ab,kw
#13 #1 or #2 or #3 or #4 or #5 or #6 or #7 or #8 or #9 or #10 or #11 or #12
#14 exp embryo transfer or exp fertilization in vitro or exp sperm injections, intracytoplasmic or exp zygote intrafallopian transfer
#15 (in Vitro adj2 fertili$).tw.
#16 (ivf or icsi or ZIFT).tw.
#17 (intracytoplas$ adj2 sperm).tw.
#18 zygote intrafallopian transfer$.tw.
#19 (embryo transfer$ or ET).tw.
#20 invitro fertili$.tw.
#21 #14 or #15 or #16 or #17 or #18 or #19 or #20
#22 #13 and #21

EMbase (Ovid)
1 "nerve stimulat*".ab,ti.
2 electro-acupuncture.ab,ti.
3 electroacupuncture.ab,ti.
4 neuro-modulation.ab,ti.
5 neuromodulation.ab,ti.
6 "trans-abdominal stimulat*".ab,ti.
7 "sacral nerve stimulat*".ab,ti.
8 "transcutaneous electr* stimulat*".ab,ti.
9 "medtronic".ab,ti.
10 "SXDZ-100".ab,ti.
11 or/1-10
12 exp fertilization in vitro/
13 exp intracytoplasmic sperm injection/
14 exp embryo transfer/
15 (in?Vitro adj2 fertili$).tw.
16 (ivf or icsi or ZIFT).tw.
17 (intracytoplas$ adj2 sperm).tw.
18 zygote intrafallopian transfer$.tw.
19 embryo transfer$.tw.
20 invitro fertili$.tw.
21 or/12-20
22 Clinical Trial/
23 Randomized Controlled Trial/
24 exp randomization/
25 Single Blind Procedure/
26 Double Blind Procedure/
27 Crossover Procedure/

28 Placebo/
29 Randomi?ed controlled trial$.tw.
30 Rct.tw.
31 random allocation.tw.
32 randomly allocated.tw.
33 allocated randomly.tw.
34 (allocated adj2 random).tw.
35 Single blind$.tw.
36 Double blind$.tw.
37 ((treble or triple) adj blind$).tw.
38 placebo$.tw.
39 prospective study/
40 or/22-39
40 11 and 21 and 40

PsycINFO (Ovid)
1 "nerve stimulat*".ab,ti.
2 electro-acupuncture.ab,ti.
3 electroacupuncture.ab,ti.
4 neuro-modulation.ab,ti.
5 neuromodulation.ab,ti.
6 "transcutaneous electr* stimulat*".ab,ti.
7 or/1-6
8 (in?Vitro adj2 fertili$).tw.
9 (ivf or icsi or ZIFT).tw.
10 (intracytoplas$ adj2 sperm).tw.
11 zygote intrafallopian transfer$.tw.
12 embryo transfer$.tw.
13 invitro fertili$.tw.
14 or/8-13
15 random.tw.
16 control.tw.
17 double-blind.tw.
18 clinical trials/
19 placebo/
20 exp Treatment/
21 or/15-20
22 7 and 14 and 21

CINAHL
S1 transcutaneous electrical nerve stimulation.
S2 ("transcutaneous electric* nerve stimulation" OR "transcutaneous nerve stimulation").ti,ab
S3 AB "nerve stimulat*"

S4 AB electro-acupuncture
S5 AB electroacupuncture
S6 AB neuro-modulation
S7 AB neuromodulation
S8 AB "transcutaneous electr* stimulat*"
S9 S1 OR S2 OR S3 OR S4 OR S5 OR S6 OR S7 OR S8
S10 TX vitro fertilisation
S11 (MM "Fertilization in Vitro")
S12 TX IVF or TX ICSI
S13 TX ovari* N3 stimulat*
S14 TX ovar* N3 hyperstimulat*
S15 TX embryo* N3 transfer*
S16 TX vitro fertilization
S17 S10 OR S11 OR S12 OR S13 OR S14 OR S15 OR S16
S18 (MH "Clinical Trials+")
S19 PT Clinical trial
S20 TX clinic* n1 trial*
S21 TX ((trebl* n1 blind*) or (trebl* n1 mask*))
S22 TX ((singl* n1 blind*) or (singl* n1 mask*)) or TX ((doubl* n1 blind*) or (doubl* n1 mask*)) or TX ((tripl* n1 blind*) or (tripl* n1 mask*)) or TX ((trebl* n1 blind*) or (trebl* n1 mask*))
S23 TX randomi* control* trial*
S24 (MH "Random Assignment")
S25 TX random* allocat*
S26 TX placebo*
S27 (MH "Placebos")
S28 (MH "Quantitative Studies")
S29 TX allocat* random*
S30 S18 OR S19 OR S20 OR S21 OR S22 OR S23 OR S24 OR S25 OR S26 OR S27 OR S28 OR S29
S31 S9 AND S17 AND S30
