# Supplementary material for: Transcutaneous electrical acupoint stimulation for pregnancy outcomes in women undergoing in vitro fertilization-embryo transfer: A systematic review and meta-analysis
Source: Front Public Health. 2022 Aug 11;10:892973. doi: 10.3389/fpubh.2022.892973 (PMC9403762; doi:10.3389/fpubh.2022.892973)
Supplement: Supplementary file 2 [file Table_2.pdf]

Supplementary Table 2 The List of Excluded Studies in the Secondary Screening.

**Author/Year**	**Title**	**Reason for exclusion**
Qu et al., 2015	Transcutaneous electrical acupoint stimulation improves outcomes of in vitro fertilization by increasing neuropeptide Y levels: A RCT	Duplicate published data
Shuai et al., 2015	Effect of transcutaneous electrical acupuncture point stimulation on endometrial receptivity in women undergoing frozen-thawed embryo transfer: a single-blind prospective randomized controlled trial	Duplicate published data
Zheng et al., 2016	Effects of transcutaneous electrical acupoint stimulation on ovarian reserve of patients with diminished ovarian reserve in IVF-ET	Duplicate published data
Gay et al., 2018	Evaluation of “Energy Resonance by Cutaneous Stimulation” Among Women Treated by In Vitro Fertilization	The interventions being studied weren’t TEAS
Zhong et al., 2017	Transcutaneous electrical acupoint stimulation for pregnancy of in vitro fertilization-embryo transfer	Non-RCT
