# Supplementary material for: Transcutaneous electrical acupoint stimulation for pregnancy outcomes in women undergoing in vitro fertilization-embryo transfer: A systematic review and meta-analysis
Source: Front Public Health. 2022 Aug 11;10:892973. doi: 10.3389/fpubh.2022.892973 (PMC9403762; doi:10.3389/fpubh.2022.892973)
Supplement: Supplementary file 3 [file Table_3.pdf]

Supplementary Table 3 The Detailed Assessment of Methodological quality of the studies using the tool RoB 2.0.

Study		Qu 2017 (29)	Shuai 2015 (20)	Zhang 2011 (18)	Zheng 2015 (19)	Feng 2013 (30)	Shuai 2019 (31)	Zhao 2019 (32)	Li 2012 (33)	Zhu 2012 (34)	Li 2012 (35)	Chen 2011 (36)	Dong 2018 (37)	Shuai 2019 (38)	Xu 2014 (39)	Fang 2021 (40)	Zhang 2018 (41)	Feng 2011 (42)	Mi 2014 (43)	Feng 2022
1.randomization process	1.1 Was the allocation sequence random?	Y	Y	Y	NI	NI	Y	NI	NI	NI	NI	NI	Y	Y	NI	Y	NI	NI	NI	Y
	1.2 Was the allocation sequence concealed until participants were enrolled and assigned to interventions?	PY	Y	NI	NI	NI	NI	NI	NI	NI	NI	NI	NI	Y	NI	NI	Y	NI	NI	Y
	1.3 Did baseline differences between intervention groups suggest a problem with the randomization process?	N	N	N	N	N	N	N	N	N	N	N	N	N	N	N	N	N	N	N

2.Deviations from the intended interventions	2.1. Were participants aware of their assigned intervention during the trial?	Y	PN	PN	NI	Y	Y	Y	PN	Y	Y	Y	Y	PN	Y	PN	Y	Y	Y	Y
	2.2. Were carers and people delivering the interventions aware of participants' assigned intervention during the trial?	PN	Y	Y	NI	Y	Y	Y	PY	Y	Y	Y	Y	Y	Y	PY	Y	Y	Y	Y
	2.3. [If applicable:] If Y/PY/NI to 2.1 or 2.2: Were important nonprotocol interventions balanced across intervention groups?	PY	PY	PY	PY	PY	PY	PY	PY	PY	PY	PY	PY	PY	PY	PY	PY	PY	PY	PY

2.4. [If applicable:] Were there failures in implementing the intervention that could have affected the outcome?	PN	N	PN	PN	PN	N	N	PY	N	N	N	N	N	N	PN	N	N	N	N
2.5. [If applicable:] Was there non-adherence to the assigned intervention regimen that could have affected participants' outcomes?	N	N	PN	PN	PN	N	N	PY	N	N	N	N	N	N	PN	N	N	N	N

	2.6. If N/PN/NI to 2.3, or Y/PY/NI to 2.4 or 2.5: Was an appropriate analysis used to estimate the effect of adhering to the intervention?	NA	NA	NA	NA	NA	NA	NA	PY	NA	NA	NA	NA	NA	NA	NA	NA	NA	NA	NA
3.Missing outcome data	3.1 Were data for this outcome available for all, or nearly all, participants randomized?	PY	Y	PY	PY	PN	Y	Y	PY	Y	Y	PN	PY	PY	Y	PY	PN	PN	PN	Y
	3.2 If N/PN/NI to 3.1: Is there evidence that the result was not biased by missing outcome data?	NA	NA	NA	NA	N	NA	NA	NA	NA	NA	N	NA	NA	NA	NA	N	N	N	NA

	3.3 If N/PN to 3.2: Could missingness in the outcome depend on its true value?	NA	NA	NA	NA	PN	NA	NA	NA	NA	NA	PN	NA	NA	NA	NA	PN	PN	PN	NA
	3.4 If Y/PY/NI to 3.3: Is it likely that missingness in the outcome depended on its true value?	NA	NA	NA	NA	NA	NA	NA	NA	NA	NA	NA	NA	NA	NA	NA	NA	NA	NA	NA
4.Measurement of the outcome	4.1 Was the method of measuring the outcome inappropriate?	N	N	N	N	N	N	N	N	N	N	N	N	N	N	N	N	N	N	N

4.2 Could measurement or ascertainment of the outcome have differed between intervention groups?	N	N	N	N	N	N	N	N	N	N	N	N	N	N	N	N	N	N	N
4.3 If N/PN/NI to 4.1 and 4.2: Were outcome assessors aware of the intervention received by study participants?	N	PN	PN	NI	NI	NI	NI	NI	NI	NI	NI	NI	NI	NI	NI	NI	NI	NI	PN

4.4 If Y/PY/NI to 4.3: Could assessment of the outcome have been influenced by knowledge of intervention received?	NA	NA	NA	N	N	N	N	N	N	N	N	N	N	N	N	N	N	N	NA
4.5 If Y/PY/NI to 4.4: Is it likely that assessment of the outcome was influenced by knowledge of intervention received?	NA	NA	NA	NA	NA	NA	NA	NA	NA	NA	NA	NA	NA	NA	NA	NA	NA	NA	NA

5.Selection of the reported result	5.1 Were the data that produced this result analysed in accordance with a pre-specified analysis plan that was finalized before unblinded outcome data were available for analysis?	PY	PY	PY	PY	PY	PY	PY	PY	PY	PY	PY	PY	PY	PY	PY	PY	PY	PY	PY

5.2. Is the numerical result being assessed likely to have been selected, on the basis of the results, from multiple eligible outcome measurements (e.g. scales, definitions, time points) within the outcome domain?	N	N	N	N	N	N	N	N	N	N	N	N	N	N	N	N	N	N	N
5.3 Is the numerical result being assessed likely to have been selected, on the basis of the results, from multiple eligible analyses of the data?	PN	PN	PN	PN	PN	PN	PN	PN	PN	PN	PN	PN	PN	PN	PN	PN	PN	PN	PN

Notes: Y, yes; PY, probably yes; PN, probably no; N, no; NI, no information; NA, not applicable.
