# Supplementary material for: Transcutaneous electrical acupoint stimulation for pregnancy outcomes in women undergoing in vitro fertilization-embryo transfer: A systematic review and meta-analysis
Source: Front Public Health. 2022 Aug 11;10:892973. doi: 10.3389/fpubh.2022.892973 (PMC9403762; doi:10.3389/fpubh.2022.892973)
Supplement: Supplementary file 4 [file Table_4.pdf]

Supplementary Table 4. Characteristics of the Included RCTs.

Study	Participants						Intervention	Comparator	Outcomes	Conclusions (+/-)
	Infertility reasons	Number (I/C)	Age (years) (I/C)	BMI(kg/m^2) (I/C)	Duration of infertility	IVF-ET protocol				
Qu 2017 (29)	Gamete transport disorders	(121/119/ 121)/120	((31.22±5.92)/(30.44±5.82)/ (31.16±6.09))/(29.81±6.17)	((22.97±6.59)/ (21.77±5.98)/ (23.14±6.55))/ (21.53±6.28)	((5.84±3.99)/(6.30 ±4.01)/(5.92±4.49))/(6.49±4.17)	GnRH agonist long protocol	3 intervention groups (TEAS-2Hz group, TEAS-100Hz group, and TEAS-2/100Hz group); 2 sessions: ①24h before TVOR, 30mins at SP10(Xuehai), SP8(Diji), LR3(Taichong), and ST36(Zusanli); ②2h before ET, 30mins at EX-CA1(Zigong), RN4(Guanyuan), PC6(Neiguan) and RN12(Zhongwan).	No TEAS group: no TEAS.	CPR;LBR;	TEAS using a frequency of 2/100 Hz could help to improve the IVF outcomes.(+)
Shuai 2015 (20)	NR	34/34	(29.47±3.24)/(29.65±2.60)	(21.99±2.71)/(2 2.32±1.64)	(4.56±3.25)/ (3.88±2.29)	HCG-induc ed natural cycle	TEAS group: CV3(Zhongji), CV4(Guanyuan), SP6(Sanyinjiao) and EX-CA1(Zigong) (dispersed-dense waves, 2Hz, 10-20mA) at 3 menstrual cycles prior to FET and 6 times per cycle on alternate days starting on M3 of each cycle,30mins/day.	Mock TEAS group: intermittent 2Hz (10s on and 20s off) TEAS at an intensity of 5mA, and the rest are the same as the TEAS group.	CPR;LBR	In patients undergoing FET, TEAS may have beneficial effects on endometrial HOXA10 expression and ultrasound markers of endometrial receptivity. These findings may explain the improvement in clinical outcome of FET associated with the use of TEAS.(+)

Zhang 2011 (18)	NR	(110/110)/110	((32.60±4.90)/(31.90±5.30))/(31.50±5.20)	((22.6±3.4)/(23.2±3))/(22.6±3.5)	NR	NR	2 intervention groups (double TEAS group, and single TEAS group); 2 sessions: ①24h before ET, 30mins at SP8(Diji), ST29(Guilai), EX-CA1(Zigong) and SP10(Xuehai) (2Hz, 15-20mA); ②30mins after ET, 30mins at ST36(Zusanli) and KI3(Taixi), BL23(Shenshu) and RN4(Guanyuan) (2Hz, 10-12mA). Double TEAS group:①②; Single TEAS group:②.	Mock TEAS group: intermittent 2Hz (10s on and 20s off, 5mA), and the rest are the same as the single TEAS group.	CPR;LBR; BPR	TEAS, especially double TEAS, significantly improved the clinical outcome of ET.(+)

Zheng 2015 (19)	Ovulation dysfunction	60/(60/ 60)	(36.05±5.48)/((36.88±4.65)/(36. 95±4.34))	(24.14±4.32)/ ((23.56±2.63)/ (23.32±2.85))	(4.44±2.98)/((4.75 ±2.64)/(5.25±3.23))	GnRH agonist short protocol	TEAS group: RN4(Guanyuan), RN3(Zhongji), SP6 (Sanyinjiao), EX-CA1(Zigong), ST25(Tianshu), BL23(Shenshu), DU3(Yaoyangguan), and DU4(Mingmen) (2Hz, 20-25mA) at 3 menstrual cycles prior to ovulation cycle and continued during the ovulation cycle until the TVOR day, everyday, 30mins/day.	2 control groups (mock TEAS group, and no TEAS group). Mock TEAS group: intermittent electric current (3s on and 7s off, 5mA), and the rest are the same as the TEAS group. No TEAS group: no TEAS.	CPR;EMR;HQER	TEAS treatment could improve the CPR in patients with DOR during IVF and ET cycles.(+)

Feng 2013 (30)	NR	45/45	(31.21±0.66)/(30.1±0.77)	(22.18±0.40)/ (22.51±0.35)	(3.18±0.36)/ (3.9±0.39)	NR	TEAS group: 2 sessions: ①24h before ET, 30mins at SP10(Xuehai), EX-CA1(Zigong), SP8 (Diji), and ST29(Guilai)(2Hz, 20-25mA); ②0.5h-2h after ET, 30mins at RN12(Zhongwan), Shenshu(BL23), KI3(Taixi), RN4(Guanyuan), and ST36(Zusanli)(2Hz, 8-15mA).	No TEAS group: no TEAS.	CPR;BPR	TEAS could improve the ovarian function of patients and improve the therapeutic effect of IVF.(+)

Shuai 2019 (31)	NR	30/30	(35.9±3.1)/(36.1±2.6)	NR	(4.9±3.5)/ (5.0±3.2)	GnRH agonist short protocol	TEAS group: RN4(Guanyuan), ST25(Tianshu), BL23(Shenshu), DU4(Mingmen), DU3(Yaoyangguan), EX-CA1(Zigong), and SP6(Sanyinjiao) (2Hz, 20-25mA), from Gn initiation day to hCG day, 20mins per time, 3 times/week.	No TEAS group: no TEAS.	CPR;HQER	TEAS can improve the endometrial receptivity, and then enhance the embryonic planting rate and CPR in patients with frozen-thawed ET.(+)

Zhao 2019 (32)	Ovulation dysfunction	43/43	(30.69±3.59)/(30.72±3.56)	(27.33±2.91)/ (26.65±2.79)	(3.92±2.11)/ (3.36±1.41)	GnRH agonist long protocol	TEAS group: RN4(Guanyuan), RN3(Zhongji), SP6(Sanyinjiao), and EX-CA1(Zigong), from Gn initiation day to hCG day, 30mins per time, every other day for 1 time.	Mock acupuncture group: making patients feel that the needle had penetrated the skin but didn't pierce the epidermis actually. The time, frequency and acupoints are the same as those of the TEAS group.	CPR;BPR;HQER	The mechanism of improving oocyte quality in infertility patients with PCOS is related to regulating ovarian granulosa cell Wnt pathway, upregulating the expression of key factor Cx43 protein in the pathway, promoting granulosa cell proliferation, improving ovarian microenvironment and follicular membrane blood flow.(+)

Li 2012 (33)	NR	62/60	(31.31±0.59)/(30.95±0.67)	(22.06±0.30)/ (22.34±0.27)	(3.32±0.3)/ (4.1±0.36)	NR	TEAS group: 2 sessions: ①24h before ET, 30mins at SP10(Xuehai), EX-CA1(Zigong), SP8 (Diji), and ST29(Guilai) (2Hz, 20-25mA); ② 0.5h-2h after ET, 30mins at RN12(Zhongwan), BL23(Shenshu), KI3(Taixi), RN4(Guanyuan), and ST36(Zusanli) (2Hz, 8-15mA).	Mock TEAS group: operation is the same as the TEAS group with the mock Han's device.	CPR;BPR; EMR	TEAS can improve IVF/ICSI-ET or frozen-thawed ET on pregnancy rate.(+)

Zhu 2012 (34)	Ovulation dysfunction	30/30	(35.63±1.14)/(35.91±0.91)	(21.86±0.29)/ (22.11±0.28)	(4.24±0.39)/ (4.31±0.28)	GnRH antagonist protocol	TEAS group: RN4(Guanyuan), RN3(Zhongji), SP6(Sanyinjiao), EX-CA1(Zigong), ST25(Tianshu), BL23(Shenshu), DU3(Yaoyangguan), and DU4(Mingmen) (2Hz, 20-25mA) at 3 menstrual cycles prior to ovulation cycle and continued during the ovulation cycle until the TVOR day, everyday, 30mins/day.	No TEAS group: no TEAS.	CPR	The TEAS could modulate the hypothalamus-pituitary-ovary axis of the patients with POR or bad ovarian reserves, and improve the pregnancy rate.(+)

Li 2012 (35)	NR	45/45	(32.2±5.31)/(32.5±4.67)	(22.45±2.35)/ (22.39±2.75)	(4.13±4.56)/ (4.05±3.21)	NR	TEAS group: ST25(Tianshu), KI12(Dahe), EX-CA1(Zigong), and SP6(Sanyinjiao) (100Hz), everyday, 30mins/day. ①2 menstrual cycles before ET, from M5 to the next menstruation. ②During FET month, from the 5th day of the menstrual cycle to ovulation day.	No TEAS group: no TEAS.	CPR;BPR	TEAS can promote endometrial growth, improve endometrial receptivity, promote embryo implantation, and improve CPR.(+)

Chen 2011 (36)	Ovulation dysfunction	40/40	(36.94±5.52)/(36.52±4.41)	(22.55±2.75)/ (23.03±2.77)	(6.69±4.72)/ (6.24±3.24)	GnRH agonist short protocol	TEAS group: Guanyuan(RN4), Zhongji(RN3), Sanyinjiao(SP6), Zigong(EX-CA1), Tianshu(ST25), Shenshu(BL23), Yaoyangguan(DU3), and Mingmen(DU4) (2Hz, 20-25mA), from the first day after menstruation to the day after LH peak, and at 100Hz, 15-20mA from the second day after LH peak to the next menstruation at 3 menstrual cycles prior to IVF-ET, and at 2Hz, 20-25mA from the first day after menstruation to the hCG day during COH, everyday, 30mins/day.	No TEAS group: no TEAS.	CPR;HQER	The TEAS could modulate the hypothalamus-pituitary-ovary axis and be beneficial to pregnancy outcome of the patients with POR.(+)

Dong 2018 (37)	NR	42/41	(32.0±5.0)/(31.0±4.0)	(22.6(18.4,25.4))/(21.3(18.7,27.5))	(3(1,15))/(2(1,10))	GnRH antagonist protocol	TEAS group: from M2/3 to the ET day (2Hz, 20-25mA), everyday, 30mins/day. Menstrual period: DU4(Mingmen), and Ex-B13(Shiqizhui); Period after menstruation and before ovulation: BL17(Geshu), BL23(Shenshu), KI3(Taixi), SP6(Sanyinjiao); Ovulatory period: EX-CA1(Zigong), RN6(Qihai), ST36(Zusanli) RN4(Guanyuan), and KI7(Fuliu); Premenstrual period: RN4(Guanyuan), RN6(Qihai), LR3(Taichong), and GB34(Yanglingquan).	No TEAS group: no TEAS.	CPR;BPR;HQER	The efficacy of TEAS sequential therapy combined with western medicine antagonist promotion program on the infertile patients undergoing IVF-ET is better than western medicine alone, especially in reducing the dosage of promoting drugs and improving the rate of excellent embryos.(+)

Shuai 2019 (38)	NR	61/61	(31.23±3.78)/(31.58±3.07)	(22.01±1.81)/ (22.39±2.87)	(5.09±2.78)/ (5.71±3.59)	GnRH agonist long protocol	TEAS group: RN4(Guanyuan), RN3(Zhongji), SP6(Sanyinjiao), and EX-CA1(Zigong) (dense-disperse frequency wave, 2Hz, 9-25mA), on alternate days from day 5 of the COH up to the ET day, 30mins/day.	Mock TEAS group: intermittent 2Hz (10s on and 10s off, 5mA), and the rest are the same as the TEAS group.	CPR;LBR	TEAS significantly improves the clinical outcomes of subsequent IVF cycles among women who have experienced RIF.(+)

Xu 2014 (39)	NR	82/94	(32.5±4.6)/(31.9±4.3)	(21.92±3.27)/ (21.65±3.26)	(5.1±3.7)/ (4.8±3.6)	Natural cycle/ hormonally controlled cycle	TEAS group: ① from M10 to 2 days before ET day, ST25(Tianshu), RN4(Guanyuan), RN3(Zhongji), EX-CA1(Zigong), and SP6(Sanyinjiao)(100Hz, 20-25mA), everyday, 30mins/day.② 24h before ET, 30mins at SP10(Xuehai), EX-CA1(Zigong), SP8(Diji), and ST29(Guilai) (2Hz, 20-25mA); ③ 0.5h after ET, 30mins at RN12 (Zhongwan), BL23(Shenshu), KI3(Taixi), RN4 (Guanyuan), and ST36 (Zusanli) (2Hz, 8-15mA).	No TEAS group: no TEAS.	CPR;BPR	TEAS can improve endometrial receptivity in patients with RIF, thus increasing implantation rate in frozen-thawed ET cycle.(+)

Fang 2021 (40)	NR	100/100	(31.36±3.37)/(31.21±3.37)	(23.63±2.96)/ (23.53±3.54)	(4.51±1.94)/ (4.85±2.05)	GnRH agonist long protocol	TEAS group: RN4(Guanyuan), RN3(Zhongji), SP6(Sanyinjiao), EX-CA1(Zigong), ST25(Tianshu), BL23(Shenshu), DU3(Yaoyangguan), and DU4(Mingmen) (2Hz, 20-25mA) at 2 menstrual cycles prior to COH, everday, 30mins/day.	Mock TEAS group: intermittent electric current (3s on and 7s off), and the rest are the same as the TEAS group.	CPR;LBR;HQER	TEAS treatment up-regulated the levels of BDNF in serum and follicular fluid, reduced Gn dosage in IVF cycle, and elevated the fertilization rate and high-quality embryo rate. Elevated BDNF level might be important factor for TEAS to improve IVF pregnancy outcomes.(+)
Zhang 2018 (41)	NR	(585/394)/728	((31.4±4.3)/(31.8±4.1))/ (30.5±4.3)	((21.70±2.86)/ (21.70±3.08))/ (21.60±2.80)	((4.7±3.7)/ (4.4±3.6))/ (4.4±3.3)	Luteal phase modulation protocol	2 intervention groups (A group, and B group).A group: 2 sessions: ①24h before ET, 30mins at SP8(Diji), EX-CA1(Zigong) and Sp10 (Xuehai)(2Hz); ②2h after ET, 30mins at ST36(Zusanli) and KI3(Taixi), RN12(Zhongwan) and RN4(Guanyuan)(2Hz). B group: 9 sessions: on the basis of A group, ③from 2nd day to 8th day after ET, 30mins at PC6(Neiguan), KI3(Taixi), RN12(Zhongwan) and RN4(Guanyuan)(2Hz), everday, once a day.	No TEAS group: no TEAS.	CPR;LBR; EMR	Our study preliminarily suggested that TEAS treatment would have the efficacy in improving pregnancy rate in IVF-ET.(+)

Feng 2011 (42)	NR	(120/120)/(120/120/120)	((32±5.0)/(32±5.2))/((32±5.2)/(32±5.0)/(31±4.8))	((22.6±3.4)/(23.2±3.0))/((22.6±3.5)/(23.1±3.1)/(22.0±3.4))	NR	NR	2 intervention groups (double TEAS group, and single TEAS group). 2 sessions: ①24h before ET, 30mins at SP8(Diji), ST29(Guilai), EX-CA1(Zigong) and SP10 (Xuehai) (2Hz, 15-20mA); ②30mins after ET, 30mins at ST36(Zusanli) and KI3(Taixi), BL23(Shenshu) and RN4(Guanyuan) (2Hz, 10-12mA).Double TEAS group:①②; Single TEAS group:②.	3 control groups (mock TEAS-1 group, mock TEAS-2 group, and no TEAS group).<br>Mock TEAS-1 group: with true acupoints, 30mins after ET, 30mins at ST36(Zusanli) and KI3(Taixi), BL23(Shenshu) and RN4(Guanyuan) (5mA);<br>Mock TEAS-2 group: with mock acupoints, 30mins after ET, 30mins at RN12(Zhongwan), RN13(Shangwan) and ST40(Fenglong) (5mA);<br>No TEAS group: no TEAS.	CPR;BPR;LBR;EMR	TEAS on the day of ET significantly improves the reproductive outcome of IVF/ICSI, compared with no treatment and placebo treatment.(+)

Mi 2014 (43)	Ovulation dysfunction	32/32	(38±0.92)/(37.8±0.55)	(23.11±0.73)/ (24.31±0.53)	(4.31±0.6)/ (4.75±0.58)	GnRH agonist short protocol	TEAS group: RN4(Guanyuan), RN3(Zhongji), SP6(Sanyinjiao), EX-CA1(Zigong), ST25(Tianshu), BL23(Shenshu), DU3(Yaoyangguan), and DU4(Mingmen) (2Hz, 20-25mA) at 3 menstrual cycles prior to COH and continued during the COH until the TVOR day, everday, 30mins/day.	No TEAS group: no TEAS.	CPR;HQER	The TEAS could modulate the hypothalamus-pituitary-ovary axis of the patients with ovarian low response, improve ovary blood flow and ovarian reaction, increase the number of retrieved oocytes so as to improve the high grade oocyte rate.(+)

Feng 2022	NR	362/369	(31.5±4.4)/(31.7±4.7)	(22.5±3.0)/ (22.4±3.1)	(3(2,6))/(4(2,6))	NR	TEAS group: 2 sessions: ①24h before ET, 30mins at SP10(Xuehai), EX-CA1(Zigong), SP8 (Diji), and ST29(Guilai) (2Hz, 10mA or 15-20mA according to the tolerability); ②0.5h after ET, 30mins at RN12(Zhongwan), BL23(Shenshu), KI3(Taixi), RN4(Guanyuan), and ST36(Zusanli) (2Hz, 10mA initially and increased gradually according to the tolerability).	No TEAS group: no TEAS.	CPR;LBR;BPR; EMR;BDR	TEAS significantly improved the CPR. It might be due to improved endometrial receptivity. (+)

Notes: NR, not referred; POR, poor ovarian response; DOR, decreased ovarian reserve; PCOS, polycystic ovary syndrome; COH, controlled ovarian hyperstimulation; I, intervention; C, control; ICSI, intracytoplasmic sperm injection; ET, embryo transfer; IVF, in-vitro fertilization; TVOR, trans-vaginal oocyte retrieval; TEAS, transcutaneous electrical acupoint stimulation; h, hours; mins, minutes; s, seconds; M, menstruation; RIF, recurrent implantation failure; Gn, gonadotropins; hCG, human chorionic gonadotropin; CPR, clinical pregnancy rate; LBR, live birth rate; BPR, biochemical pregnancy rate; HQER, high-quality embryo rate; BDR, birth defects rate; FET, frozen-thawed embryo transfer; LH, luteinizing hormone; +, positive; -, negative.
